# New Appliances and Things Medical

**Published:** 1902-09-27

**Authors:** 


### NEW APPLIANCES AND THINGS MEDICAL.

[We shall be glad to receive at our Office, 28 & 29 Southampton Street, Strand, London, W.C., from the manufacturers, specimens of all new preparations and appliances which may be brought out from time to time.]

### ANTIPHLOGISTINE.

(Denver Chemical Manufacturing Company, 18 Jeffreys Road, Clapham Road, London, S.W.)

Antiphlogistine is a hygroscopic paste for application to inflamed surfaces. It is composed of glycerine, boric acid, salicylic acid, iron carbonate, menthol, eucalyptus, and iodine, combined with dehydrated silicate of alumina and magnesia. The therapeutic properties of these chemical substances, individually and combined, are such as are indicated in cases of local inflammation for the restoration of an impeded circulation, for the removal of pain and the promotion of the normal processes of nutrition. In practice this paste is proved to be very comfortable to the patient, and highly efficacious in the treatment of indolent ulcers and acute inflammatory conditions. Antiphlogistine is a very elegant preparation and skilfully combined; it is spread on the skin like gelatine and zinc pastes, and can be readily peeled off after it has completed its work.

### FONTALIS: AN ENGLISH TABLE-WATER.

(Camwal Limited, 45 Gifford Street, London, N.)

Fontalis is the name of new table-water of exceptional purity which the Camwal Co. are introducing to the public. The water is obtained from a deep boring on the Camwal estate at Harrogate. In his analytical report Professor Percy F. Frankland states that the mineral matter which it contains is almost exclusively composed of alkaline chlorides and carbonate, and practically free from salts of lime and magnesia. The water from this spring is therefore well suited for table-purposes, and by careful aëration and bottling on the part of the proprietors it is rendered one of the most reliable and delicious aërated waters with which we are acquainted. It has the further merit also of being a British production.

